# Pediatric Papillary Renal Cell Carcinoma in a Horseshoe Kidney: A Case Report with Review of the Literature

**DOI:** 10.1155/2015/841237

**Published:** 2015-08-02

**Authors:** Abelardo Loya-Solis, Lucía Alemán-Meza, Luis Carlos Canales-Martínez, Rodolfo Franco-Márquez, Alim Adriana Rincón-Bahena, Karla María Nuñez-Barragán, Raquel Garza-Guajardo, Marco Antonio Ponce-Camacho

**Affiliations:** ^1^Pathology Department, Spanish Hospital of Mexico, Ejercito Nacional 613, 11560 Mexico City, DF, Mexico; ^2^Pathology Department, University Hospital “Dr. Jose E. Gonzalez” and Medical School of the Autonomous University of Nuevo Leon, Francisco I. Madero and Gonzalitos, 64460 Monterrey, NL, Mexico; ^3^Medical Imaging Department, University Hospital “Dr. Jose E. Gonzalez” and Medical School of the Autonomous University of Nuevo Leon, Francisco I. Madero and Gonzalitos, 64460 Monterrey, NL, Mexico

## Abstract

Renal cell carcinoma is the most common malignancy of the kidney in adults. In children, however, it only accounts for an estimated 1.8 to 6.3% of all pediatric malignant renal tumors. Papillary renal cell carcinoma is the second most common type of renal cell carcinoma in children. We present the case of a 12-year-old boy with a 2-month history of abdominal pain, unexplained weight loss, and gross hematuria. Computed tomography revealed a horseshoe kidney and a well-defined mass of 4 cm arising from the lower pole of the right kidney. Microscopically the tumor was composed of papillae covered with cells with abundant eosinophilic cytoplasm and high-grade nuclei with prominent nucleoli. Immunohistochemistry was performed; EMA, Vimentin, and AMACR were strongly positive while CK7, CD10, RCC antigen, TFE3, HMB-45, and WT-1 were negative. Currently, 10 months after the surgical procedure, the patient remains clinically and radiologically disease-free.

## 1. Introduction

Malignant neoplasms of the kidney are a fairly common group of tumors representing up to 2% of the total human cancer burden [1]. Renal cell carcinoma (RCC) is the most common malignancy of the kidney in adults [1]. In children, however, it only accounts for an estimated 1.8 to 6.3% of all pediatric malignant renal tumors [2, 3]. Papillary renal cell carcinoma (PRCC) is a malignant renal parenchymal tumor with a papillary or tubulopapillary architecture. PRCC is the second most common type of RCC in children and accounts for 30% of RCCs [4]. The usual clinical presentation consists of abdominal pain, palpable mass, and gross hematuria, but this classic triad is only found in 6% of cases [5]. No proper therapy has been defined for children with RCC. Surgery constitutes the main treatment and results in cure when the tumor is localized and completely resected [6].

## 2. Case Report

**Figure 1:**
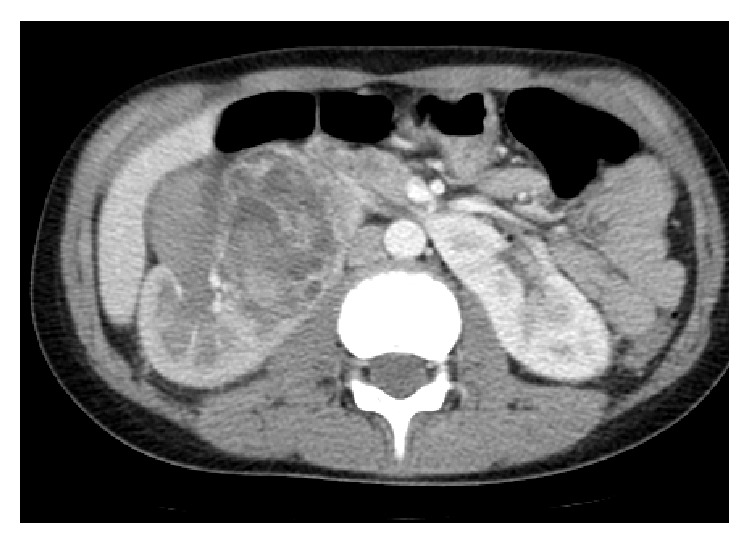
Abdominal computed tomography revealed an ill-defined tumor arising from the lower pole of the right kidney.

**Figure 2:**
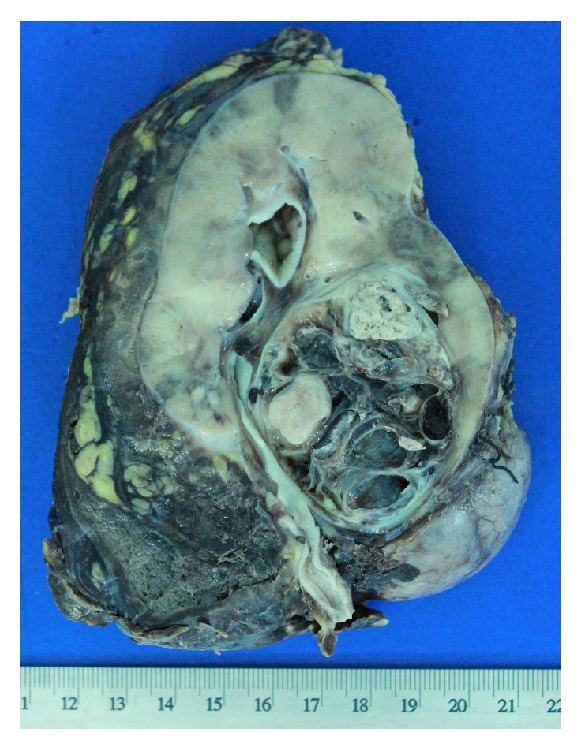
Gross photograph of the cut surface of the right kidney showing a heterogeneous tumor mass.

**Figure 3:**
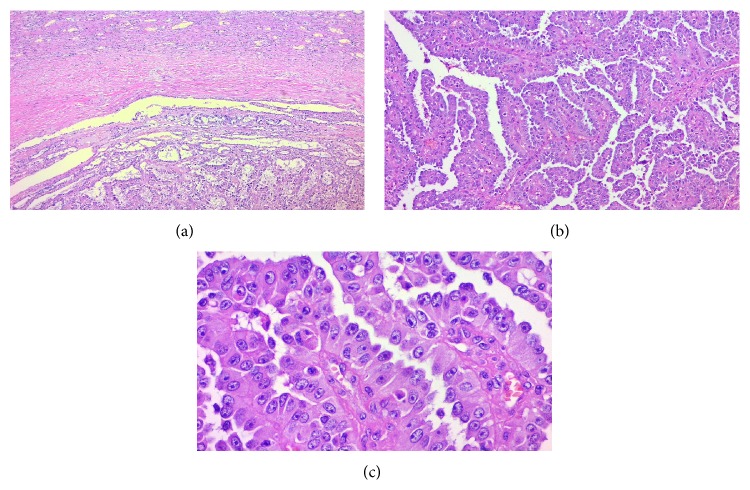
(a) Tumor pseudocapsule composed of fibrous tissue. H&E stain, ×50. (b) Papillae covered by large cells with abundant eosinophilic cytoplasm. H&E stain, ×100. (c) Pseudostratified high-grade nuclei with prominent nucleoli. H&E stain, ×400.

**Figure 4:**
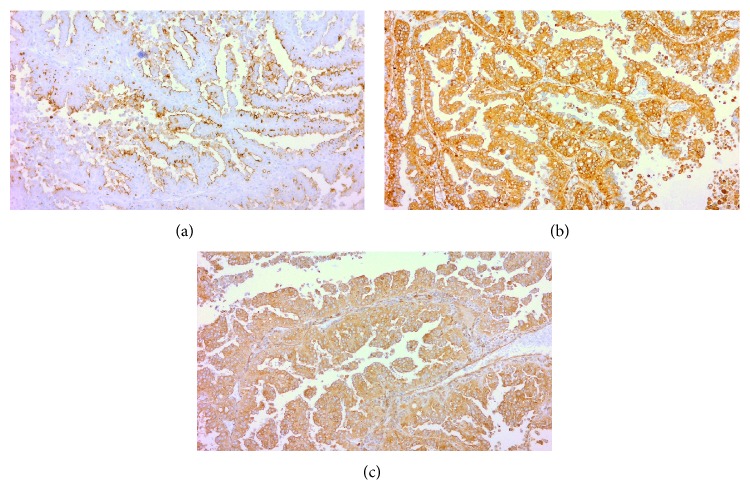
(a) Reactivity to EMA. Immunohistochemical stain with anti-EMA antibody, ×100. (b) Reactivity to Vimentin. Immunohistochemical stain with anti-Vimentin antibody, ×100. (c) Reactivity to AMACR. Immunohistochemical stain with anti-AMACR antibody, ×100.

A 12-year-old boy presented with a 2-month history of abdominal pain, unexplained weight loss, and gross hematuria. Physical examination revealed no abnormalities. Urine examination confirmed gross hematuria and urine culture was sterile. Laboratory blood analyses were within normal limits and urinary excretion of catecholamines was also within the normal range. Upper abdominal computed tomography revealed a horseshoe kidney and a well-defined mass of 4 cm arising from the lower pole of the right kidney (Figure 1). Right nephroureterectomy with division of the isthmus of the horseshoe kidney was performed. The cut surface of the excised specimen showed a solid, circumscribed, heterogeneous tumor (size 4.3 × 3.5 cm), with solid areas alternating with cystic and hemorrhagic areas with widely clear margins (Figure 2). Histopathological examination revealed a tumor surrounded by a pseudocapsule, composed of papillae covered with cells with abundant eosinophilic cytoplasm and high-grade nuclei with prominent nucleoli (Figure 3). Immunohistochemistry was performed using cytokeratin 7 (CK7), epithelial membrane antigen (EMA), Vimentin, CD10, RCC antigen, TFE3, HMB-45, WT-1, and alpha-methylacyl coenzyme-A racemase (AMACR). EMA, Vimentin, and AMACR were strongly positive (Figure 4). CK7, CD10, RCC antigen, TFE3, HMB-45, and WT-1 were negative. In view of these histopathological and immunohistochemistry findings a type 2 PRCC was diagnosed. Currently, 10 months after the surgical procedure, the patient remains clinically and radiologically disease-free.

## 3. Discussion

Horseshoe kidney is the most common type of renal fusion anomaly; it occurs in 1 per 400–600 live births and is twice as common in males as in females [7]. Horseshoe kidney consists of two distinct functioning kidneys on each side of the midline, connected at the lower poles by an isthmus of functioning renal parenchyma or fibrous tissue that crosses the midline of the body [8]. Almost one-third of all patients with horseshoe kidney remain asymptomatic, only being diagnosed incidentally during physical examination or by CT scans [9]. The incidence of neoplasms in a horseshoe kidney is approximately 3 to 4 times greater than in normal population and is possibly the result of chronic obstruction, lithiasis, and infection [3]. RCC is the most common neoplasm described in horseshoe kidneys, accounting for about 50% of cases [10]. The tumor can be localized at any part of the kidney but is most frequently found within the isthmus [11]. There has been only one reported case of a PRCC arising in a horseshoe kidney [9], and this is, to the best of our knowledge, the first reported case in a child.

The differential diagnosis of a renal tumor with tubulopapillary architecture in a child, like in our case, should include translocation-associated RCCs (Xp11.2 translocation RCC and RCC with t(6; 11)), epithelial predominant Wilms tumor (EPWT), metanephric adenoma (MA), and PRCC.

Unlike other types of RCC, Xp11.2 translocation RCC is not defined by its histologic features, although the most common feature is a papillary or nested architecture made up of cells with ample acidophilic cytoplasm. This tumor does not express cytokeratins or EMA, but HMB-45, CD10, and RCC are often expressed [12]. Nuclear immunoreactivity for TFE3 is confirmatory of this entity [12]. RCC with t(6; 11) features a biphasic population of neoplastic cells. The main cell type is epithelioid, with abundance of clear to eosinophilic cytoplasm and round nuclei with small nucleoli. The second population is composed of smaller cells typically clustered around nodules of hyaline basement membrane material [13]. Most cases have been negative for cytokeratins and EMA but positive for HMB-45 and Melan A [14]. Nuclear TFEB expression by immunohistochemistry is the most commonly used technique to establish this diagnosis [15]. Our case presented negativity for HMB-45 and TFE3, allowing us to disregard the possibility of an Xp11.2 translocation RCC. Although we could not perform an immunohistochemical stain for TFEB, the lack of a second population of neoplastic cells and the negativity for HMB-45 allowed us to discard the possibility of a RCC with t(6; 11).

Both EPWT and MA have cells with little cytoplasm and low grade nuclei without nucleoli. EPWTs like PRCC present a pseudocapsule composed of fibrous tissue, while MA does not present it [16]. Both EPWT and MA are characteristically positive to WT-1 [4]. The cytological features and the lack of expression for WT-1 of our case helped us to discard EPWT and MA as possible diagnoses.

Two subtypes of PRCC are recognized based on their histologic features [17]. Type 1 PRCC is the most frequent, accounting for approximately two-thirds of all PRCCs and is composed of papillae covered with a single layer of small cells and scant clear or pale cytoplasm and uniform nuclei with inconspicuous nucleoli [18]. Type 2 PRCC is composed of tumor cells with voluminous cytoplasm and pseudostratified high-grade nuclei with prominent nucleoli [18]. These subtypes also differ in their immunohistochemical phenotypes. CK7 is positive in 87% of type 1 and 20% of type 2 lesions [19]; EMA, Vimentin, and AMACR are typically positive in both types [4]. The tumor in our case was diagnosed as a type 2 PRCC due to its morphological features, lack of expression for CK7, and positivity for EMA, Vimentin, and AMACR. Due to the age of our patient and the type of RCC he presented, it is important to mention an autosomal dominant syndrome known as hereditary leiomyomatosis and renal cell cancer, in which early age at onset and type 2 PRCCs are common [20]. In this syndrome patients also develop multiple cutaneous leiomyomas in almost all cases and definitive diagnosis is only made by the presence of a germline mutation in the fumarate hydratase gene [21]. Our patient, however, did not present any cutaneous nodules on physical examination nor did he have a family history of this syndrome.

Not much is known about treatment and outcome of the different subtypes of childhood RCC. It appears to have a similar stage-for-stage outcome to RCC in adults [22], but more data is needed to establish this. Neither chemotherapy nor radiation therapy have demonstrated significant activity in adult or pediatric patients with metastatic or residual RCC, regardless of the histologic type. For this reason, adjuvant therapy is not currently recommended for children with PRCC and no residual tumor [4]. Concerning the outcome of a patient with a tumor in a horseshoe kidney, presenting the anomaly does not seem to affect prognosis [10].
